# The impact of treatment with antidepressants on HBA1c- and LDL levels in type 2 diabetes: A real-world within-subject study

**DOI:** 10.1192/j.eurpsy.2021.631

**Published:** 2021-08-13

**Authors:** C. Rohde, R.W. Thomsen, S. Østergaard

**Affiliations:** 1 Department Of Affective Disorders, Aarhus University Hospital, Århus N, Denmark; 2 Department Of Clinical Epidemiology, Aarhus University Hospital, Århus N, Denmark; 3 Department Of Clinical Medicine, Aarhus University Hospital, Århus N, Denmark; 4 Department Of Clinical Medicine, Aarhus University, Aarhus, Denmark

**Keywords:** Depression, Type 2 Diabetes, Glycemic control, Population-based study

## Abstract

**Introduction:**

With regard to glycemic control in type 2 diabetes (T2D), treatment with antidepressant drugs is a double-edged sword. Real-world, population-based data on the impact of antidepressant treatment on glycemic control in T2D is absent from the literature.

**Objectives:**

To estimate the impact of treatment initiation or termination with an antidepressant on HbA_1c_ levels in individuals with T2D.

**Methods:**

Population-based, within-subject, study design examining HbA_1c_ levels in the 16 months leading up to - and the 16 months following - antidepressant treatment initiation or termination, respectively. All individuals with newly developed T2D between 1 January 2000 and 31 October 2016 were identified. Study population 1 consisted of individuals that initiated antidepressant treatment after incident T2D and age- and sex matched individuals with T2D and without antidepressant treatment. Study population 2 consisted of individuals with prevalent antidepressant use at the time of incident T2D, who terminated antidepressant treatment during follow-up, and age- and sex matched individuals with T2D and without antidepressant treatment.

**Results:**

Antidepressant treatment initiation was associated with a decrease in HbA_1c_ levels (7.05% to 6.89%). The age- and sex matched individuals did not have a change in mean HbA_1c_ levels after the matched date. Antidepressant treatment termination was associated with a decrease in HbA_1c_ levels (7.05% to 6.73%). Age- and sex matched individuals did not see a change in HbA_1c_ levels after the matched date.

**Conclusions:**

These findings suggest that antidepressant treatment initiation is not associated with adverse effects with regard to glycemic control in T2D. Rather, the data are indicative of a beneficial effect.

**Conflict of interest:**

Aarhus University funded the study. CR was supported by the Danish Diabetes Academy, funded by the Novo Nordisk Foundation, grant number NNF17SA0031406. The funders had no role in the study design, data analysis, interpretation of data, or writing of the

